# Supramolecular assembly of pyrene–DNA conjugates: influence of pyrene substitution pattern and implications for artificial LHCs†

**DOI:** 10.1039/d3ob01375h

**Published:** 2023-09-21

**Authors:** Jan Thiede, Simon Rothenbühler, Ioan Iacovache, Simon M. Langenegger, Benoît Zuber, Robert Häner

**Affiliations:** a Department of Chemistry, Biochemistry, and Pharmaceutical Sciences, University of Bern Freiestrasse 3 CH-3012 Bern Switzerland robert.haener@unibe.ch https://www.haener.dcbp.unibe.ch; b Institute of Anatomy, University of Bern Baltzerstrasse 2 CH-3012 Bern Switzerland

## Abstract

The supramolecular self-assembly of pyrene–DNA conjugates into nanostructures is presented. DNA functionalized with different types of pyrene isomers at the 3′-end self-assemble into nano-objects. The shape of the nanostructures is influenced by the type of pyrene isomer appended to the DNA. Multilamellar vesicles are observed with the 1,6- and 1,8-isomers, whereas conjugates of the 2,7-isomer exclusively assemble into spherical nanoparticles. Self-assembled nano-spheres obtained with the 2,7-dialkynyl pyrene isomer were used for the construction of an artificial light-harvesting complex (LHC) in combination with Cy3 as the energy acceptor.

## Abstract 2

The supramolecular self-assembly of pyrene-modified DNA into nanostructures with implications for the design and construction of artificial light-harvesting complexes is described.

## Graphical abstract

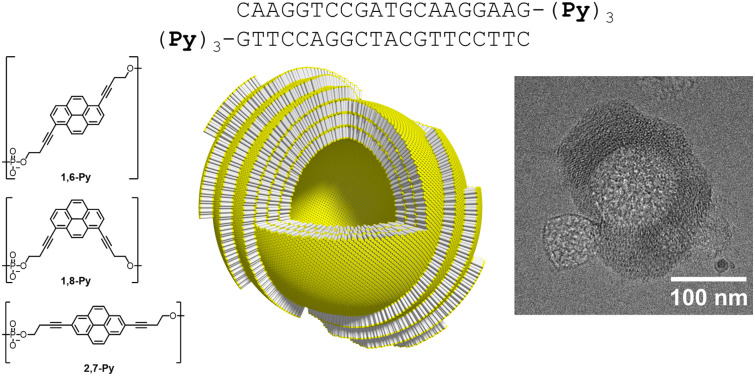

DNA nanotechnology is a rapidly evolving field of research, in which DNA is used for the assembly of well-defined nanostructures.^1–15^ The properties of the double helix allow the construction of spatially and functionally complex architectures.^16–21^ An important aspect resides in the dynamic nature of the double helix formation, which allows nanostructures to be formed in a reversible manner.^22^ DNA origami is arguably the most widely used strategy for the formation of nucleic acid nanostructures, usually involving unmodified DNA.^23–28^ The use of modified DNA, *e.g.* containing hydrophobic (sticky) ends, has been shown to be a viable alternative for the formation of DNA-based nanostructures.^29–35^ Recently, we reported the supramolecular self-assembly of 3′-end modified DNA conjugates into vesicles, using phenanthrene or tetraphenylethylene sticky ends.^36,37^ The hydrophobic interactions between the aromatic overhangs are a key element for the self-assembly process. The modified DNA duplexes self-assemble into vesicles in the presence of spermine. Spermine and other polyamines are known to facilitate DNA assembly by reducing the coulombic repulsion between the negatively charged DNA backbones.^38–40^

In this work, we describe the supramolecular self-assembly of DNA containing three pyrene units at the 3′-end (oligomers 1–6, Fig. 1). Properties such as hydrophobicity, a high molar absorption coefficient and the tendency to form exciplexes render pyrene an interesting building block for supramolecular objects.^41^ Its spectroscopic properties facilitate the monitoring of the self-assembly process and at the same time open possibilities for light-harvesting application.^42,43^ Previous publications showed that the substitution pattern in pyrene trimers can have a substantial effect on the supramolecular self-assembly.^44,45^ Here, we describe the influence of different pyrene isomers on the self-assembly of pyrene–DNA conjugates (1,6-, 1,8- and 2,7-dialkynyl derived pyrenes, Fig. 1b). Oligomers 1–6 were prepared *via* solid-phase synthesis using phosphoramidite chemistry and purified by reverse-phase HPLC according to published procedures.^37,46,47^ Cy3-modified oligonucleotide 7 was purchased commercially (Fig. 1).

**Fig. 1:**
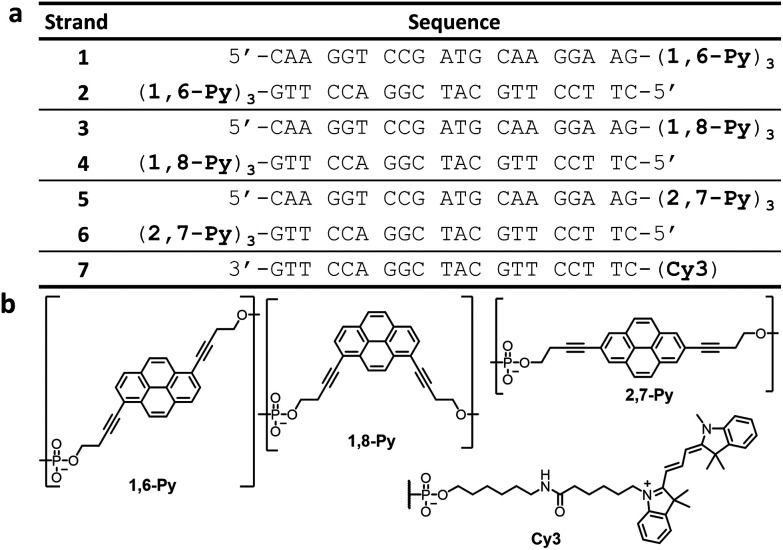
(a) Sequences of oligomers 1–7 and (b) chemical structures of 1,6-, 1,8-, and 2,7-pyrene isomers, and Cy3 modification.

Temperature dependent UV-vis spectra of hybrids 1*2, 3*4, and 5*6 were measured (Fig. 2a–c). The spectrum of 1*2 at 75 °C shows distinct absorption maxima of 1,6-dialkynyl pyrene at 365 nm and 387 nm.^44,46^ In the range from 220 nm to 320 nm, the pyrene absorption bands overlap with the ones of the DNA nucleobases. After controlled cooling (0.5 °C min^−1^, Fig. 2a) of a solution of 1*2 from 75 °C to 20 °C, the pyrene absorption bands above 320 nm exhibit a slight bathochromic shift (1–2 nm), whereas a small hypochromicity is observed for the band between 220 and 300 nm. In addition, a small degree of light scattering is observed, which indicates some aggregation of the pyrene–DNA conjugates.^41^ The absorption spectra of hybrid 3*4 (1,8-isomer, Fig. 2b) are nearly identical to the one of hybrid 1*2.^46,48^ The spectrum of the 2,7-dialkynyl pyrene–DNA conjugates (5*6) at 75 °C consists of two maxima, of which the weaker one at 342 nm originates from the pyrene units and the one at 270 nm from both, the pyrene and the DNA nucleobases (Fig. 2c).^47,49,50^ Upon cooling to 20 °C the maximum at 342 nm shifts bathochromically (2 nm), the band at 270 nm shifts hypsochromically and scattering is observed, again indicating some aggregation of the pyrene–DNA conjugates.^51^

**Fig. 2:**
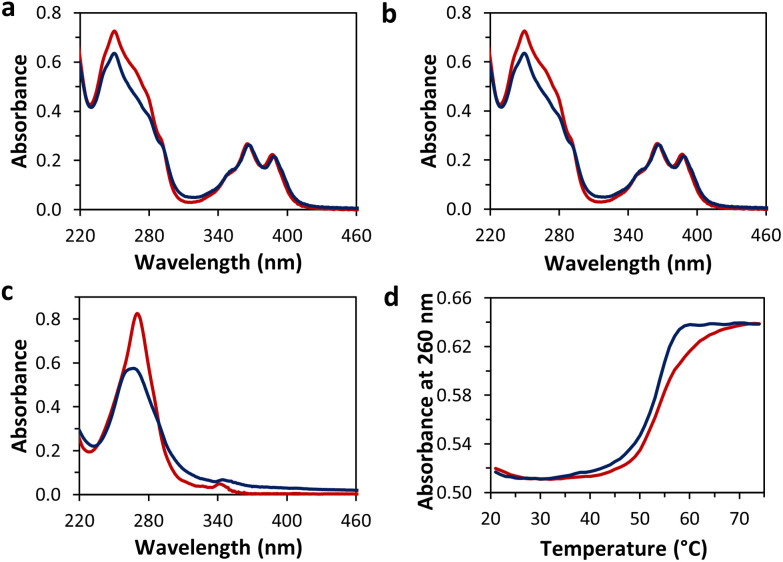
Temperature-dependent UV-vis absorption spectra of the DNA conjugates before (red, 75 °C) and after (blue, 20 °C) self-assembly: (a) 1*2, (b) 3*4, and (c) 5*6. (d) Absorbance at 260 nm during cooling from 75 °C to 20 °C (blue) and heating back to 75 °C (red) of 1*2 (gradient 0.5 °C min^−1^). Conditions: 1 μM each single strand, 10 mM sodium phosphate buffer pH 7.2, 0.03 mM spermine·4HCl, 20 vol% ethanol.

To gain additional insight into the aggregation process the absorbance was monitored at 260 nm during heating and cooling processes (Fig. 2d and Fig. S8, ESI†). Nucleation temperatures of 1*2, 3*4, and 5*6 were observed in the range from 58 to 60 °C. For all hybrids, a slight cooling/heating hysteresis was observed.

Temperature-dependent fluorescence excitation and emission spectra of the pyrene–DNA conjugates were measured (Fig. 3). Hybrids 1*2 and 3*4 exhibit pyrene excimer fluorescence with a maximum at around 525 nm.^41^ Upon cooling, a bathochromic and hypochromic shift of the emission was observed. In contrast, 5*6 show monomer (410–450 nm) in addition to excimer fluorescence (450–625 nm). Both, monomer, and excimer emission show a hyperchromic shift upon cooling.

**Fig. 3:**
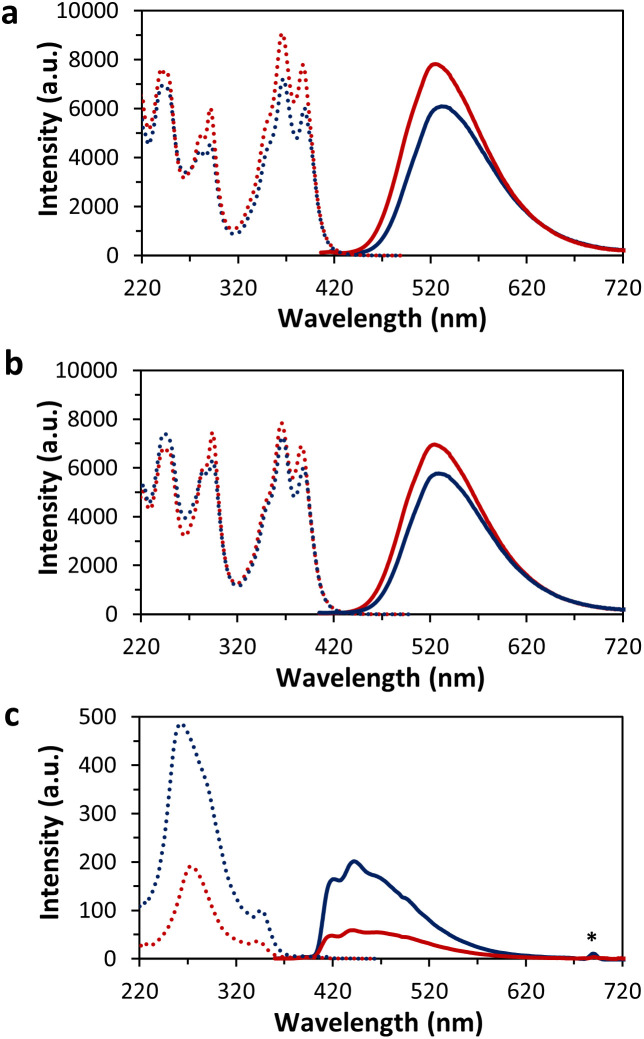
Temperature-dependent fluorescence at 75 °C (red) and 20 °C (blue), excitation (dashed) and emission spectra (solid); (a) 1*2 (*λ*_ex._ 388 nm, *λ*_em._ 525 nm), (b) 3*4 (*λ*_ex._ 388 nm, *λ*_em._ 525 nm) and (c) 5*6 (*λ*_ex._ 345 nm, *λ*_em._ 415 nm). Conditions: 1 μM each single strand, 10 mM sodium phosphate buffer pH 7.2, 0.03 mM spermine·4HCl, 20 vol% ethanol. * Second order diffraction.

Cryo-EM, AFM, and DLS provided further information on the self-assembly of the DNA conjugates into supramolecular nanostructures. Cryo-EM images of 1*2 indicate a vesicular morphology of the aggregates with a diameter of 105 ± 46 nm (Fig. 4, 5a, and Fig. S9, ESI†). For 1*2 multilamellar vesicles with an interlamellar distance of 7.5 ± 0.5 nm were observed (inset Fig. 4a). The interlamellar distance correlates well with the length of a modified DNA duplex. Therefore, we suggest that the multilamellar vesicles are formed by layers of assembled pyrene–DNA duplexes. Individual layers can interact *via* hydrophobic interactions of pyrene overhangs (Fig. 4b and c), thus leading to multilamellar vesicles. The number of lamellae can vary between different vesicles and up to 7 layers have been observed. The outer lamellae look like open shells. Also, agglomerates of several vesicles were observed in some cryo-EM images (Fig. S9, ESI†).

**Fig. 4:**
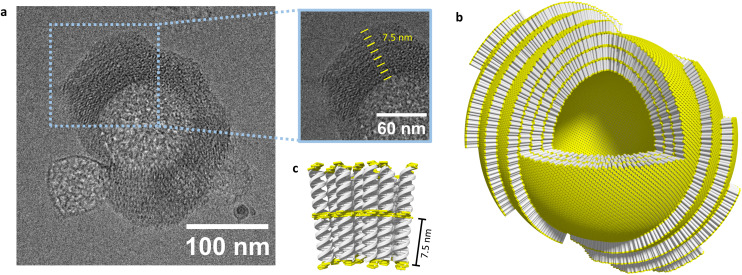
(a) Cryo-EM image of self-assembled hybrid 1*2, inset showing multilamellar arrangement, (b) schematic representation of multilamellar self-assembled vesicles, and (c) zoomed-in schematic representation of aggregated DNA strands (DNA in grey, pyrene in yellow).

**Fig. 5:**
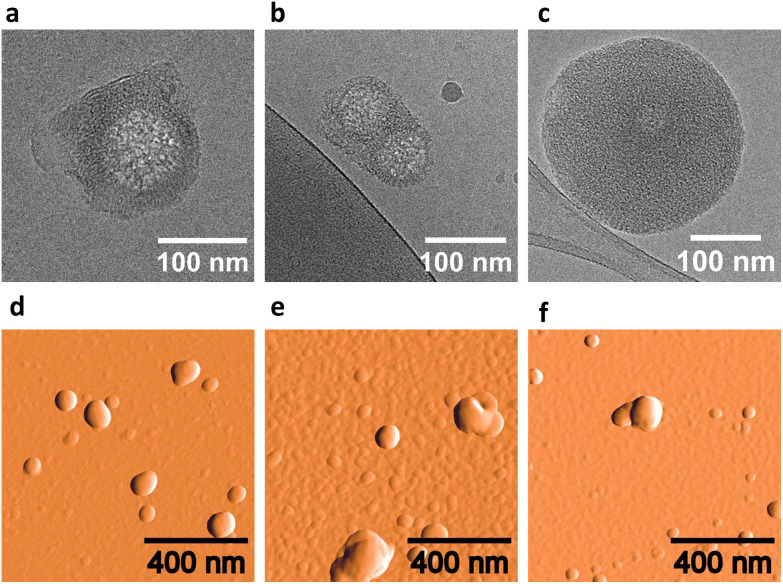
Cryo-EM images (a–c) and AFM images on APTES-modified mica (d–f) of aggregated pyrene DNA conjugates with three different pyrene isomers. From left to right: 1*2 (a and d), 3*4 (b and e), and 5*6 (c and f). Conditions: 1 μM each single strand, 10 mM sodium phosphate buffer pH 7.2, 0.03 mM spermine·4HCl, 20 vol% ethanol.

Cryo-EM images of hybrid 3*4 showed similar multilamellar vesicles with a diameter of 121 ± 29 nm, sometimes also forming groups of two or more vesicles (Fig. 5b and Fig. S10, ESI†). In contrast, cryo-EM images of 5*6 revealed spherical assemblies without a cavity (Fig. 5c and Fig. S11, ESI†). These nano-spheres have a diameter of 220 ± 60 nm. Interestingly, no agglomerates of the spheres were observed in cryo-EM images of this hybrid.

AFM measurements were performed to further investigate the morphology of the assemblies. Single and agglomerated, round shaped objects were overserved for 1*2, 3*4, and 5*6 on (3-aminopropyl)triethoxysilane (APTES) modified mica (Fig. 5d–f and Fig. S12, ESI†). The diameters of the individual vesicles 1*2 (108 ± 50 nm) and 3*4 (134 ± 47 nm) are similar. The diameter of nanostructures of 5*6 were found to be a bit larger (182 ± 55 nm). These results are in good agreement with the cryo-EM measurements.

In addition to cryo-EM and AFM, DLS experiments of the supramolecular assemblies in solution were performed. Average diameters of 192 ± 60 nm for 1*2, 196 ± 67 nm for 3*4, and 186 ± 62 nm for 5*6 were measured (Table S4 and Fig. S13, ESI†). These findings are in good agreement with the cryo-EM and AFM measurements of single, unaggregated nanostructures.

Finally, we investigated the light-harvesting properties of the self-assembled nanostructures.^52–56^ For this purpose, the complementary Cy3-modified DNA strand 7 was added to vesicles formed by 5*6 (6 mol% relative to oligomer 5; corresponds to a 1% of Cy3 per pyrene, see ESI†). Addition of 7 to vesicles formed by 5*6 leads to a minor decrease of the pyrene fluorescence and the appearance of Cy3 fluorescence at 576 nm (Fig. 6 and Fig. S15, ESI†), confirming energy transfer from the pyrenes to the Cy3. The total fluorescence quantum yield increases from 3.1% to 6.8% (Fig. 6 and Table S2, ESI†). No light-harvesting effect was observed with a non-complementary Cy3-modifed DNA strand (Fig. S14, ESI†). Energy transfer *via* FRET (Förster resonance energy transfer)^57^ has been reported for similar pyrene/Cy3 systems.^53,58^ In a pure FRET mechanism, the fluorescence of the acceptor (Cy3) cannot exceed the decrease of donor emission (pyrene). In the present system, however, the Cy3 emission largely exceeds the decrease in donor fluorescence resulting in the significant increase in the quantum yield (see above). Thus, energy transfer processes other than FRET are also involved, *i.e.* coherent energy transfer mechanisms.^59–61^

**Fig. 6:**
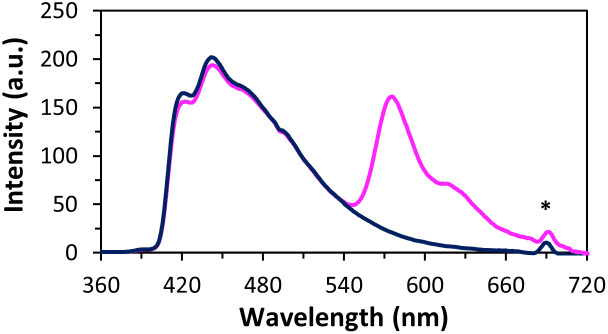
Fluorescence emission spectra of aggregated 5*6 at 20 °C (dark blue) and aggregated 5*6 after incorporation of Cy3-modifed 7 (pink, 6 mol% per 5) by reassembly at 20 °C (*λ*_ex._ 345 nm). Conditions: 1 μM 5 and 6 (with and without 0.06 μM 7), 10 mM sodium phosphate buffer pH 7.2, 0.03 mM spermine·4HCl, 20 vol% ethanol. * Second order diffraction.

In conclusion, the supramolecular self-assembly of DNA modified with pyrene sticky ends into nanostructures has been demonstrated. Three dialkynyl pyrene isomers were investigated, which all lead to the formation of vesicular assemblies with diameters between 50–300 nm as confirmed by AFM, DLS and cryo-EM. Morphologies of the assemblies were found to depend on the type of pyrene isomer. The 1,6- and 1,8-dialkynyl pyrene DNA conjugates self-organized into multilamellar vesicles while spherical aggregates were observed with the 2,7-dialkynyl pyrene isomer. Additionally, the pyrene units present within the assembled 2,7-dialkynyl pyrene–DNA conjugates act as light-harvesting antennae and transfer the energy to a Cy3-acceptor.

## Author contributions

J. T. designed the project, synthesized the oligomers, performed the experiments, analysed the data, and wrote the paper. S. R. helped in the design of the project and in the analysis of the data. I. I. performed cryo-EM experiments, analysed the data, and contributed to the writing of the paper. S. M. L. designed the project, analysed the data, created the artwork, and contributed to the writing of the paper. B. Z. designed and supervised the project and contributed to the writing of the paper. R. H. designed and supervised the project, analysed the data, and contributed to the writing of the paper.

## Conflicts of interest

There are no conflicts to declare.

## Supplementary Material

OB-021-D3OB01375H-s001
