# Supplementary material for: Supramolecular assembly of pyrene–DNA conjugates: influence of pyrene substitution pattern and implications for artificial LHCs
Source: Org Biomol Chem. 2023 Sep 21;21(39):7908–12. doi: 10.1039/d3ob01375h (PMC10566252; doi:10.1039/d3ob01375h)
Supplement: OB-021-D3OB01375H-s001 [file OB-021-D3OB01375H-s001.pdf]

Electronic Supplementary Material (ESI) for Organic & Biomolecular Chemistry.
This journal is © The Royal Society of Chemistry 2023

Electronic Supplementary Information

Supramolecular Assembly of Pyrene-DNA Conjugates: Influence of Pyrene Substitution Pattern and Implications for Artificial LHCs

Jan Thiede,[a] Simon Rothenbühler,[a] Ioan Iacovache,[b] Simon M. Langenegger,[a] Benoît Zuber,[b] and Robert Häner*[a]

[a] Department of Chemistry, Biochemistry, and Pharmaceutical Sciences, University of Bern, Freiestrasse 3, CH-3012 Bern, Switzerland

[b] Institute of Anatomy, University of Bern, Baltzerstrasse 2, CH – 3012 Bern, Switzerland

Table of contents

1. General Methods 3

2. Solid-Phase Oligomer Synthesis and Purification 4

3. Temperature-dependent UV-vis Spectra 12

4. Fluorescence Quantum Yield 12

5. Cryo-EM 13

6. AFM 15

7. Distance Measurements 16

8. DLS 17

9. Fluorescence Spectroscopy 18

10. References 18

1. General Methods

All reagents and solvents were purchased from commercial sources and used without further purification. The synthesis pyrene phosphoramidites necessary for the solid-phase synthesis of the pyrene DNA conjugates was performed according to published procedures.[1,2] The Cy3-modified DNA single strands were purchased from Microsynth (Switzerland). Water was used from a Milli-Q system. Mass spectra were measured by the Analytical Research and Services (ARS) of the University of Bern, Switzerland, on a Thermo Fisher LTQ Orbitrap XL using Nano Electrospray Ionization (NSI). All mass spectra were measured in negative ion mode in mixtures of acetonitrile/water/triethylamine. UV-vis spectra were measured on a Jasco V-730 spectrophotometer using quartz cuvettes with an optical path of 1 cm. Fluorescence spectra were collected on a Jasco spectrophotometer FP-8300 using an excitation and emission slit of 2.5 nm. The fluorescence quantum yield was determined according to published procedure[3] relative to quinine sulphate in 0.5 M H_2SO_4.[4] Supramolecular self-assembly was carried-out via thermal disassembly and reassembly. The sample solution was heated to 75 °C, then cooled with a gradient of 0.5 °C/min to 20 °C in a thermostat equipped with a Peltier. Cy3 doping experiment were conducted by addition of 6% of Cy3-modifed DNA strand to the sample solutions, followed by the integration of that strand into the aggregates by thermal disassembly reassembly (heating to 75 °C, followed by controlled cooling to 20 °C) as described above. Dynamic light scattering (DLS) experiments were performed on a Malvern Zetasizer Nano Series instrument (λ = 633 nm) in particle size distribution (PSD) mode (number value) at 20 °C. Atomic force microscopy (AFM) experiments were conducted under ambient conditions on a Nanosurf FlexAFM instrument using tapping mode. AFM samples were prepared on (3-aminopropyl)triethoxysilane (APTES)-modified mica sheets (Glimmer "V1", 20 mm x 20 mm, G250-7, Plano GmbH) according to published procedures,[5] using a sample adsorption time of 10 min. Samples for cryo-EM were plunge frozen using the FEI Vitrobot Mark 4 at room temperature and 100% humidity. In brief, copper lacey carbon grids were glow discharged (air -10 mA for 20 seconds). 3 µL of then sample were pipetted on the girds and blotted for 3 seconds before plunging into liquid ethane. Sample grids were stored in liquid nitrogen. Images were acquired using a Gatan 626 cryo holder on a Falcon III equipped FEI Tecnai F20 in nanoprobe mode. Due to the nature of the sample, acquisition settings had to be adjusted for a low total electron dose (less than 20 $e^-/Å^2$) using EPU software. Distance measurements were done in Fiji[6,7] using the multi-point tool to set marks.

2. Solid-Phase Oligomer Synthesis and Purification

Pyrene-DNA conjugates **1-6** (Table S1) were synthesized on an Applied Biosystems 394 DNA/RNA synthesizer with a 1 μmolar standard cyanoethyl phosphoramidite coupling protocol. A coupling time of 30 s was employed for both pyrene phosphoramidite and the DNA nucleobases (0.1 M in anhydrous acetonitrile). The solid-phase synthesis was started with a pyrene-modified long chain alkylamine controlled pore glass (LCAA- CPG) solid-support, which was prepared according to reported procedures.[5] After the solid-phase synthesis, **1-6** were cleaved and deprotected with aqueous NH_4OH (28-30%) at 55 °C overnight. The supernatants were collected, and the solid-support was washed three times with a solution of ethanol and Milli-Q H_2O (1:1, 3×1 mL) and the crude pyrene-DNA conjugates were lyophilized and redissolved in Milli-Q H_2O (1:1, 4ml) three times. Subsequently, the pyrene-DNA conjugates **1-6** were purified by reverse-phase HPLC (Shimadzu LC-20AT, ReproSil 100 C18, 5,0 μm, 250×4 mm) at 40 °C with a flow rate of 1 mL/min, λ: 260 nm. Solvent A: aqueous 2.1 mM triethylamine (TEA) / 25 mM 1,1,1,3,3,3-hexafluoropropan-2-ol (HFIP) pH 8; solvent B: acetonitrile; applying the gradients listed in Table S1. The purified pyrene-DNA conjugates **1-6** were dissolved in 1 ml of Milli-Q H_2O. Afterwards, the absorbance of the conjugates was measured at 260 nm to determine the concentration of the stock solutions and yields of **1-6**. The Beer-Lambert law was applied to determine the concentrations. For the DNA nucleobases and pyrene modifications the following molar absorption coefficients (at 260 nm) in $L \cdot mol^{-1} \cdot cm^{-1}$ were used: εA: 15’300; εT: 9’000; εG: 11’700; εC: 7’400, ε1,6-pyrene: 20’000; ε1,8-pyrene: 30’000; and ε2,7-pyrene: 32’000. The HPLC traces and mass spectra of **1-6** are displayed in Figure S1-S7.

Table S1 Pyrene-DNA oligonucleotide sequences **1-6**, HPLC gradients, calculated and found masses by NSI-MS, and yields.

Oligomer	Sequence (3′ to 5′)	HPLC gradient B [%] (t_R [min])	Calc. mass	found mass	Yield [%]
**1**	(**1,6-Py**)$_3$-GAA GGA ACG TAG CCT GGA AC	5 (0), 20 (24)	7400.3621	7400.3192	27
**2**	(**1,6-Py**)$_3$-GTT CCA GGC TAC GTT CCT TC	5 (0), 20 (24)	7235.2858	7235.2439	36
**3**	(**1,8-Py**)$_3$-GAA GGA ACG TAG CCT GGA AC	5 (0), 20 (24)	7400.3621	7400.3088	18
**4**	(**1,8-Py**)$_3$-GTT CCA GGC TAC GTT CCT TC	5 (0), 20 (24)	7235.2858	7235.2413	12
**5**	(**2,7-Py**)$_3$-GAA GGA ACG TAG CCT GGA AC	5 (0), 20 (24)	7400.3621	7400.3179	29
**6**	(**2,7-Py**)$_3$-GTT CCA GGC TAC GTT CCT TC	5 (0), 20 (24)	7235.2858	7235.2439	36

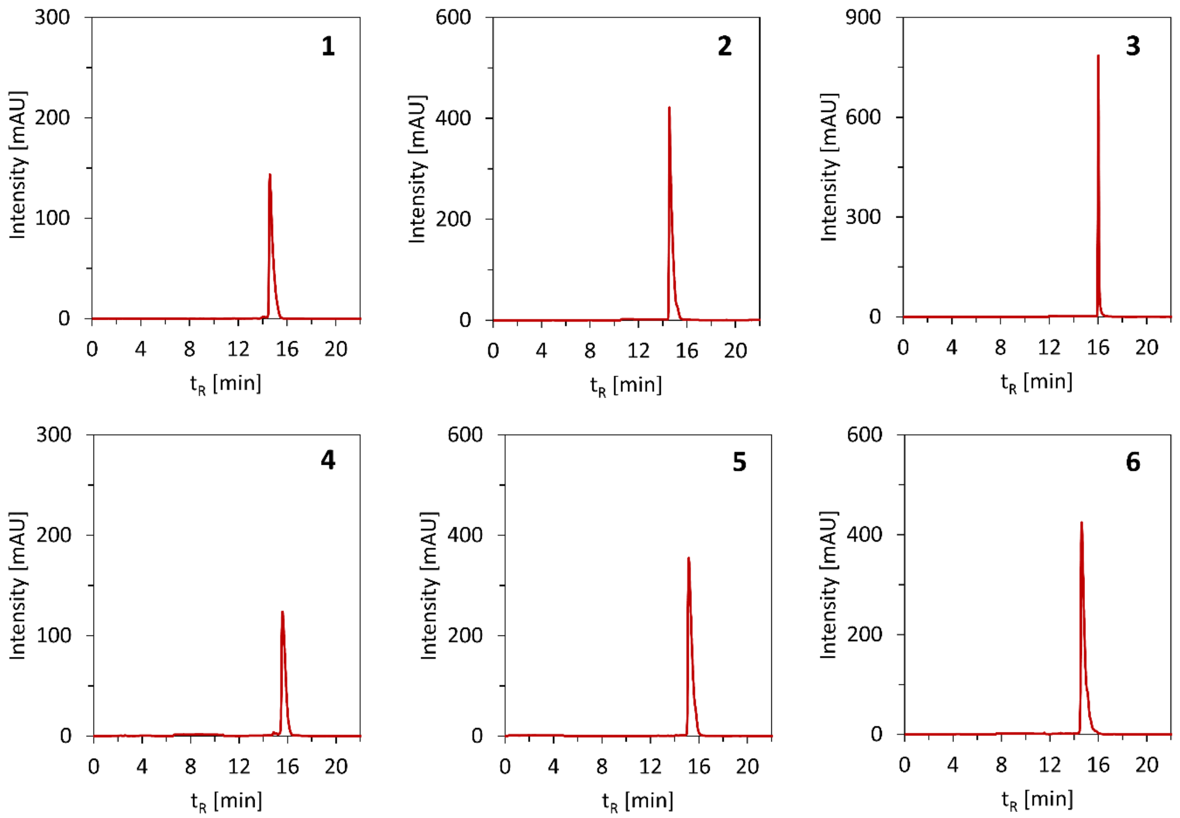

Figure S1 HPLC traces of **1-6** absorption measured at 260 nm.

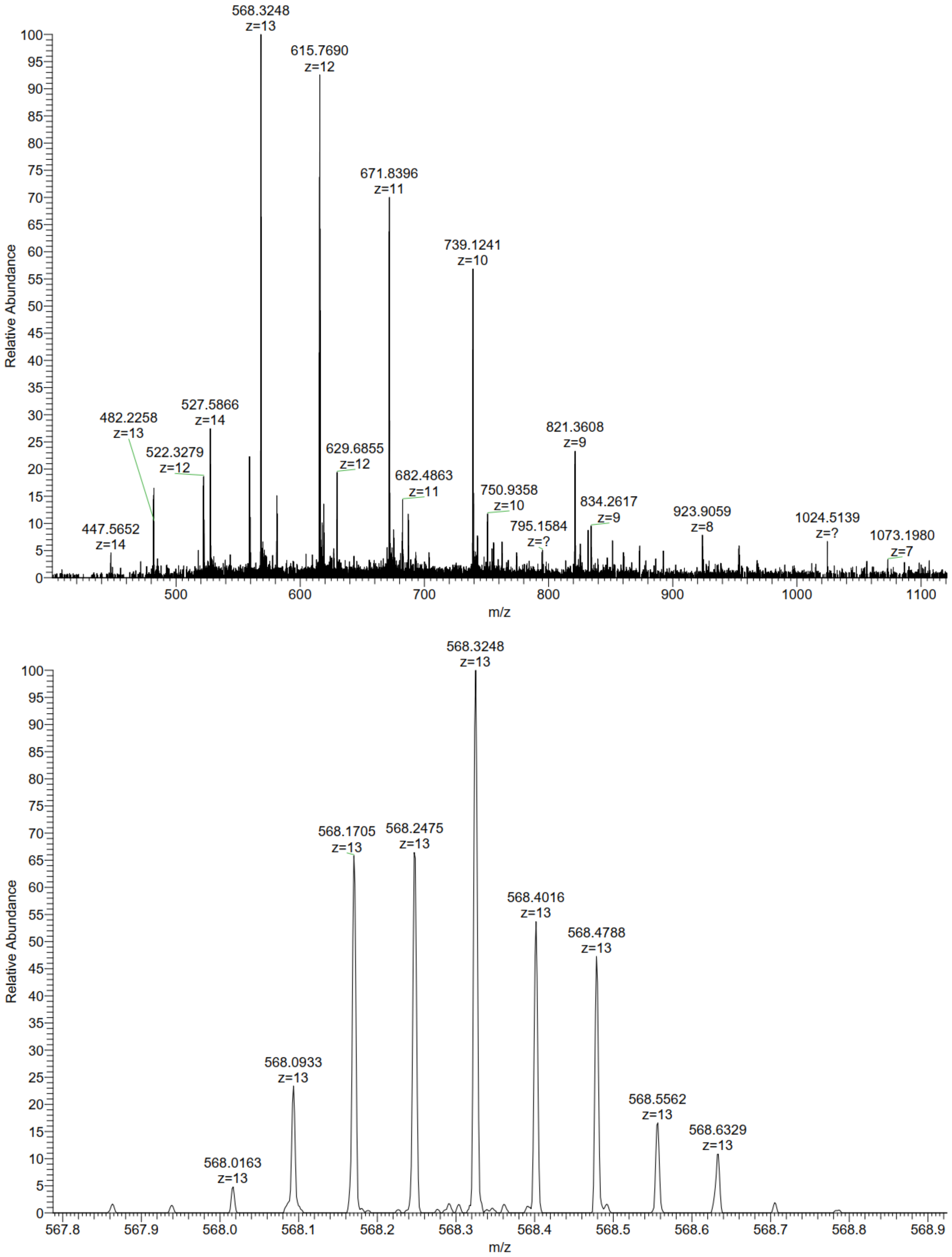

Figure S2 MS spectra of **1**.

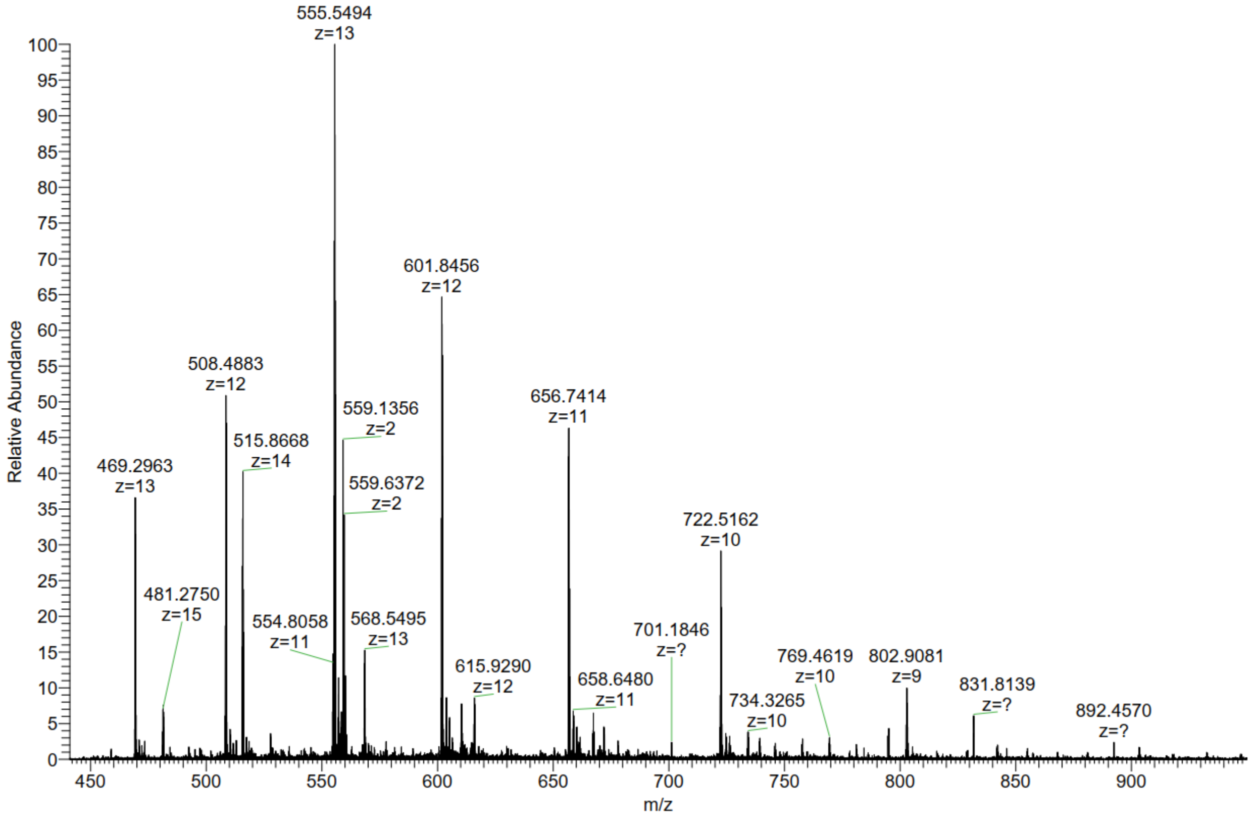

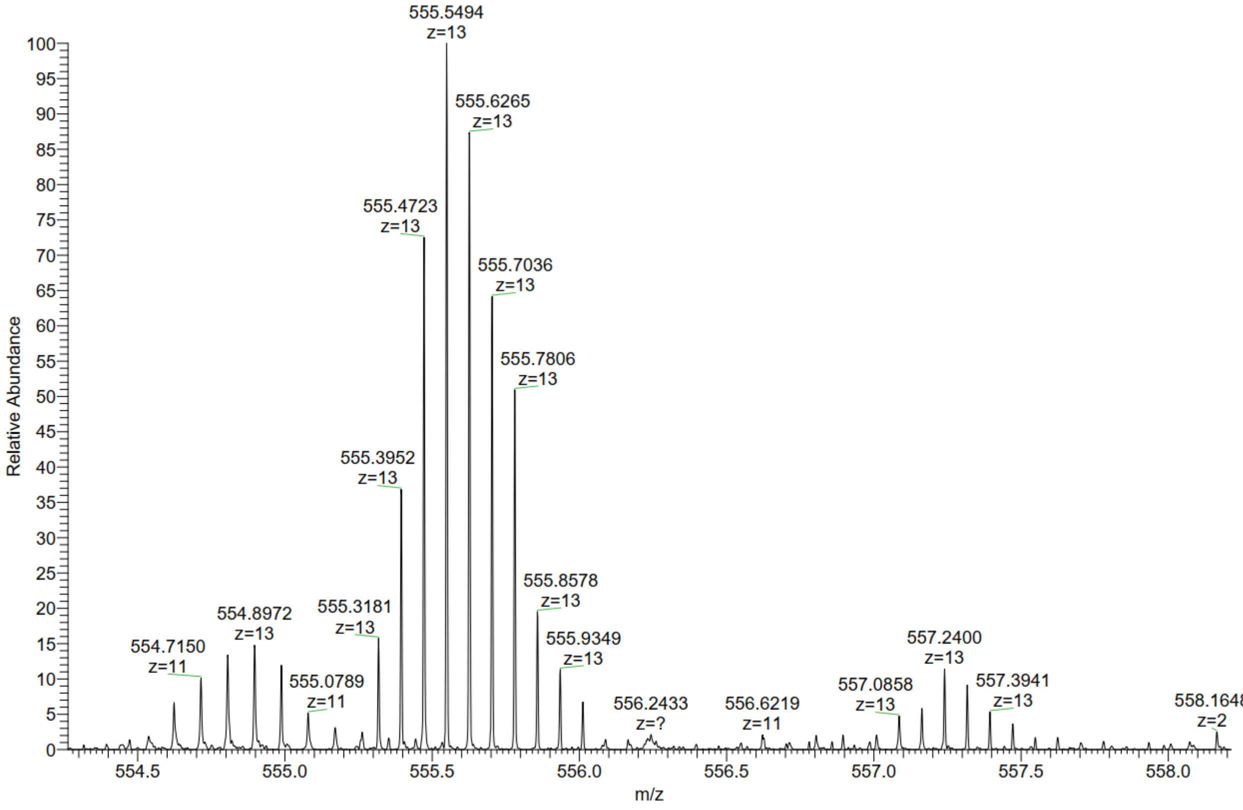

Figure S3 MS spectra of **2**.

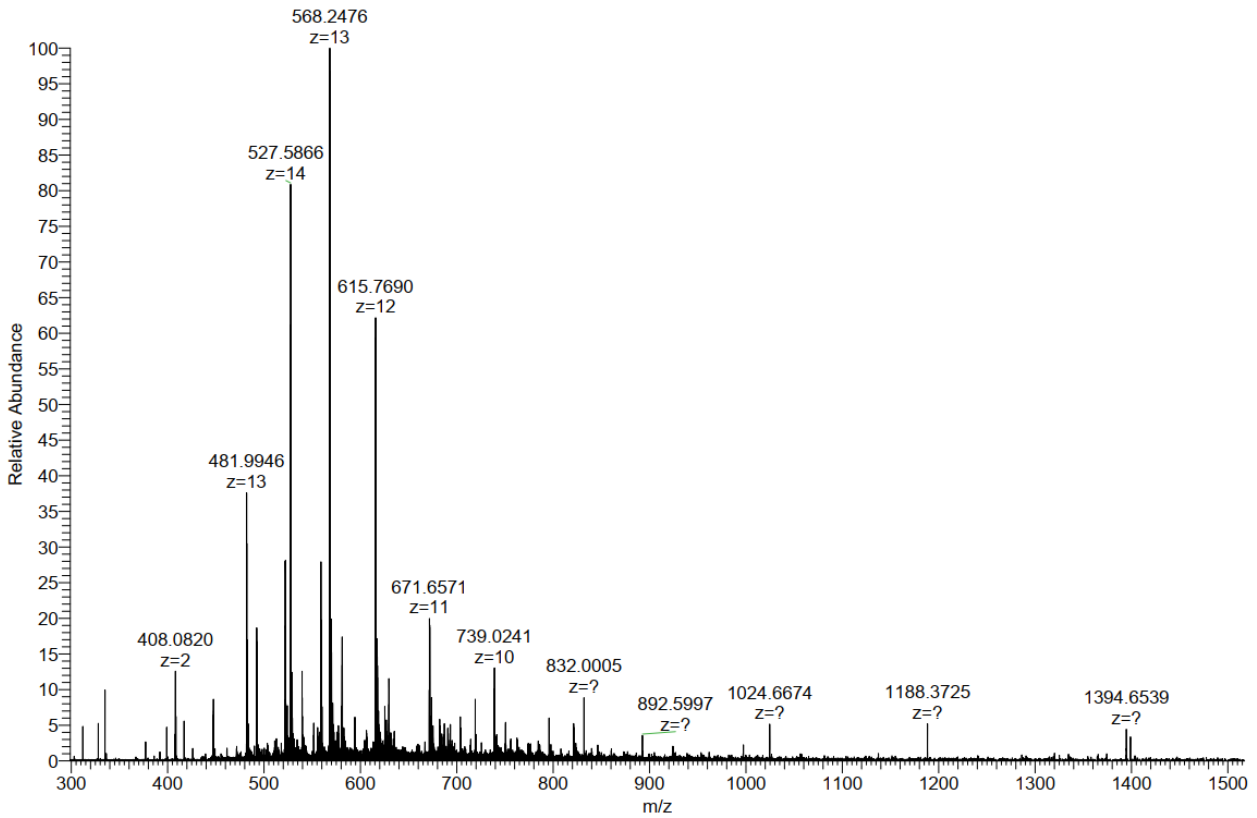

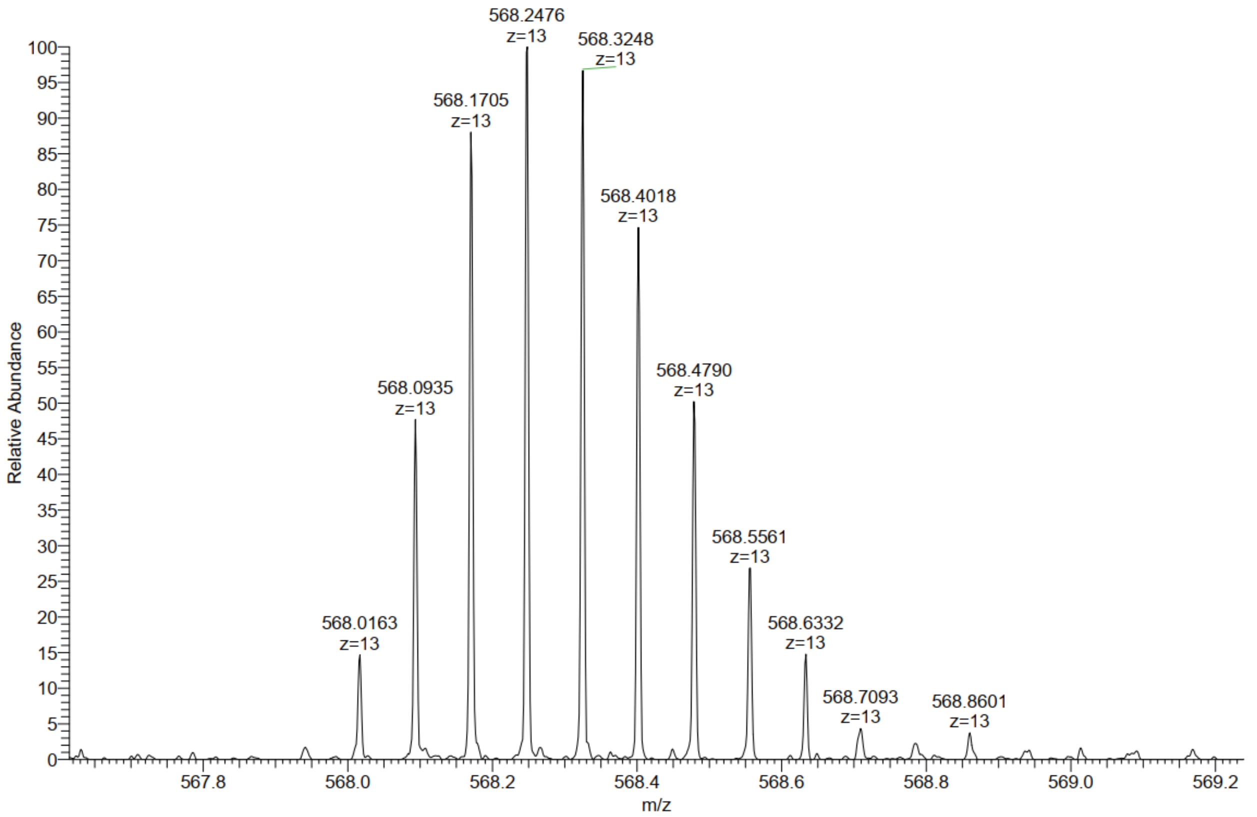

Figure S4 MS spectra of **3**.

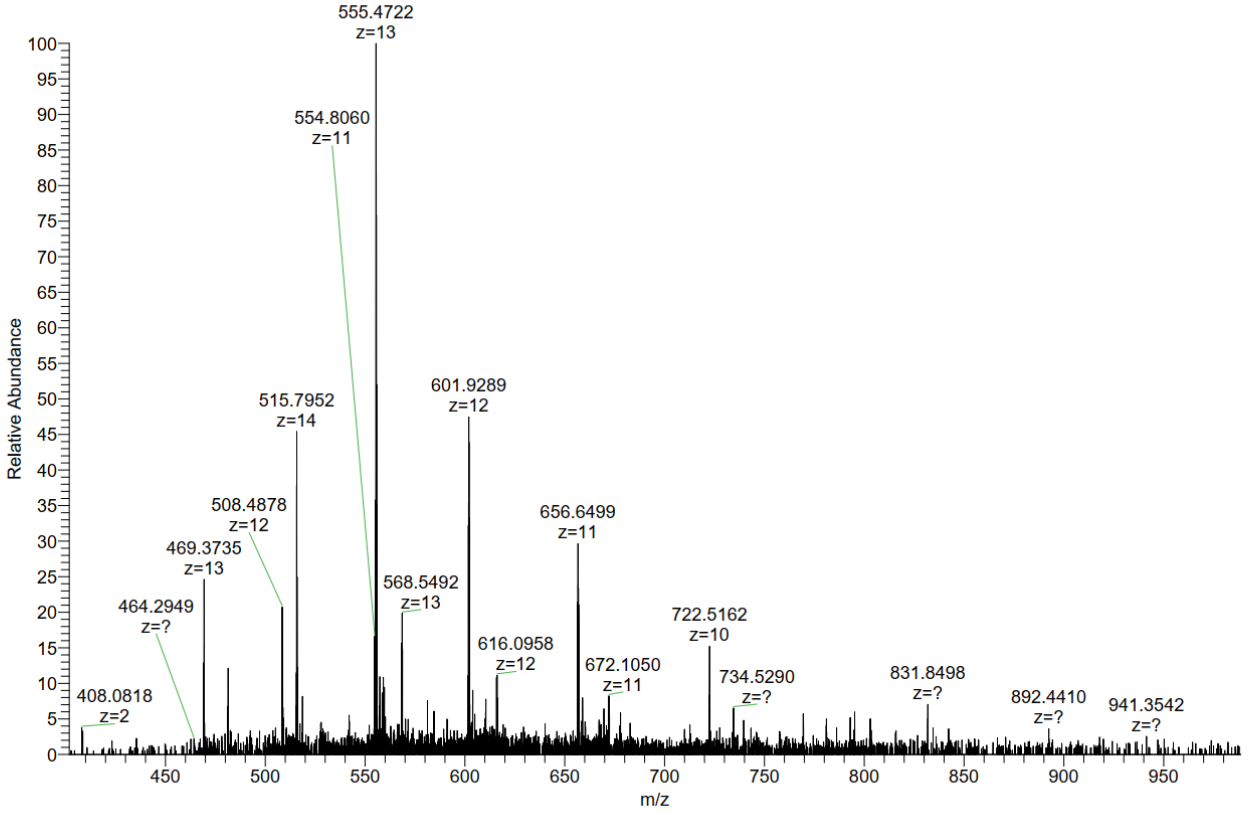

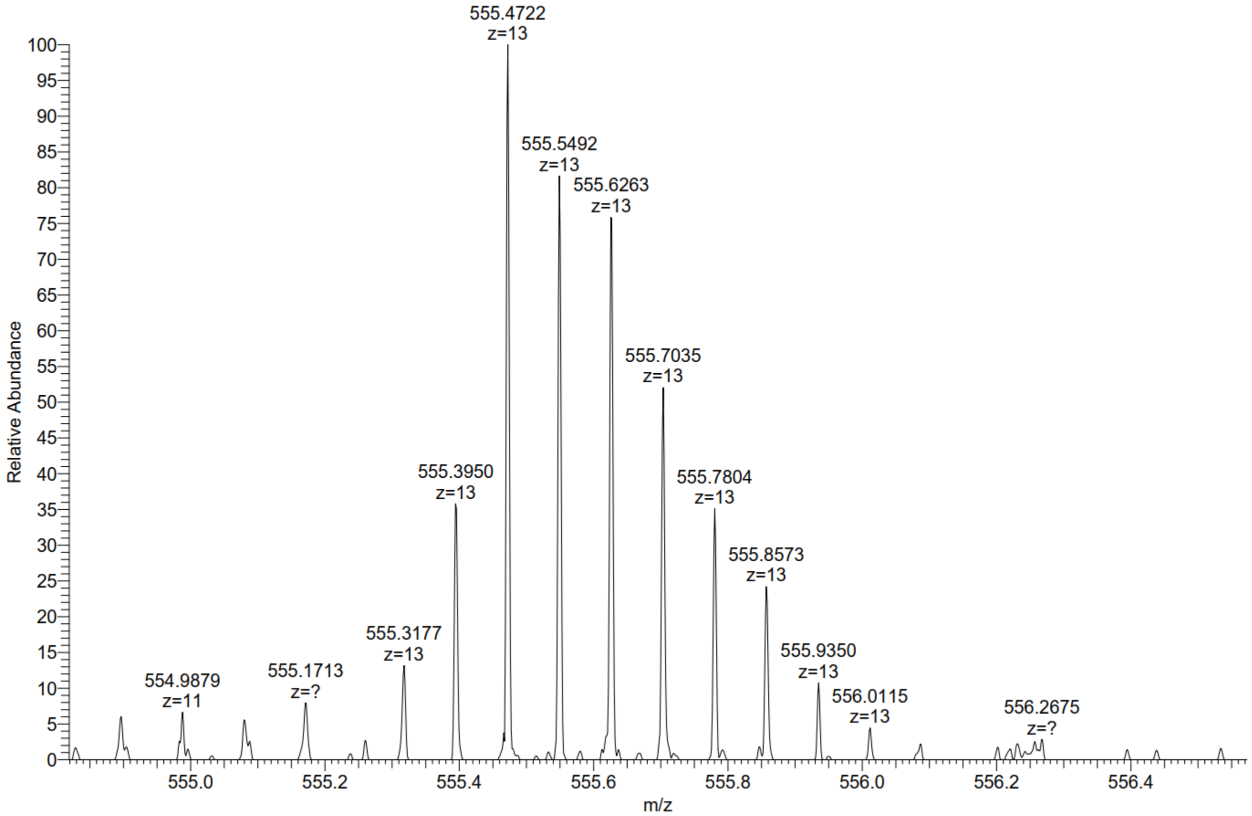

Figure S5 MS spectra of **4**.

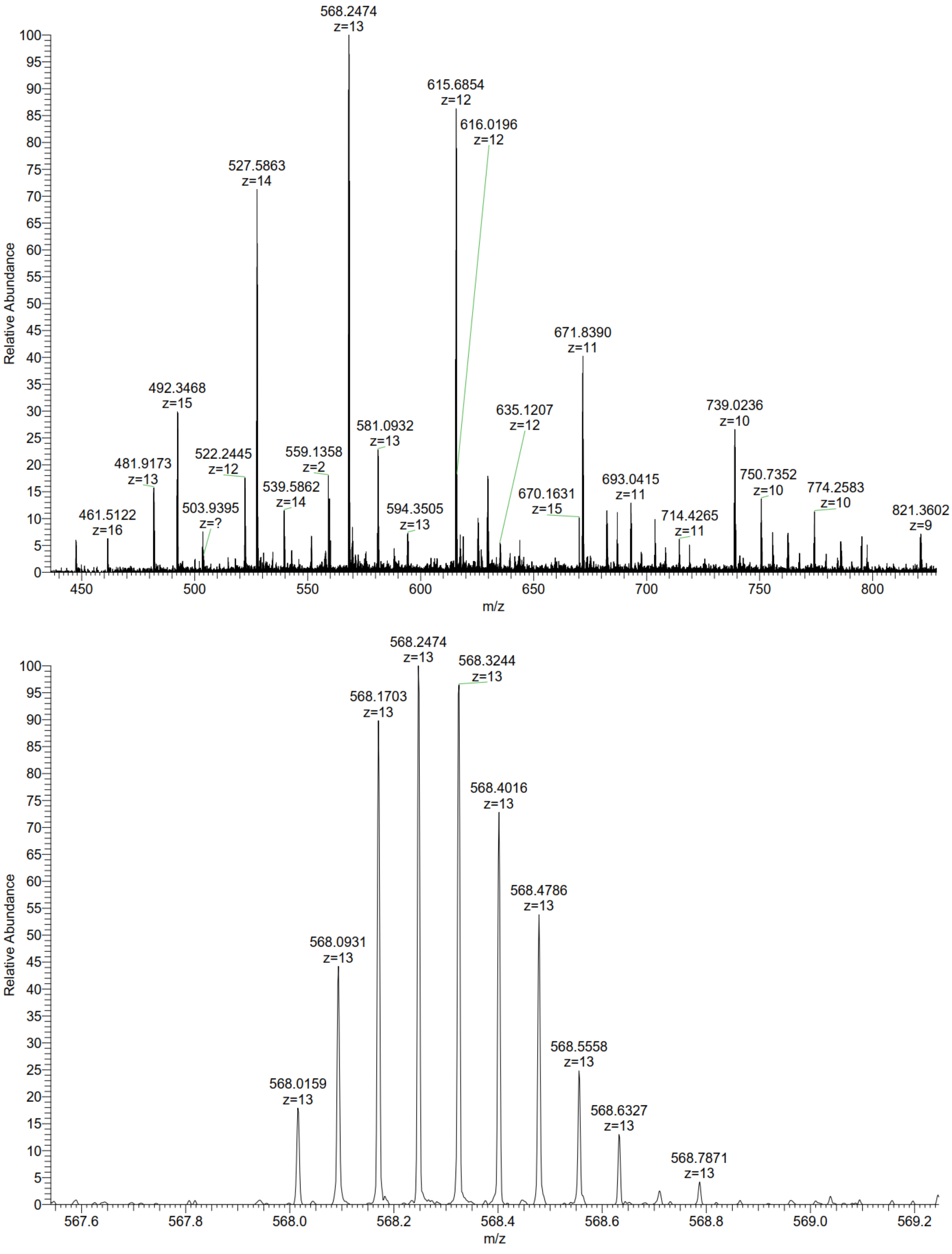

Figure S6 MS spectra of **5**.

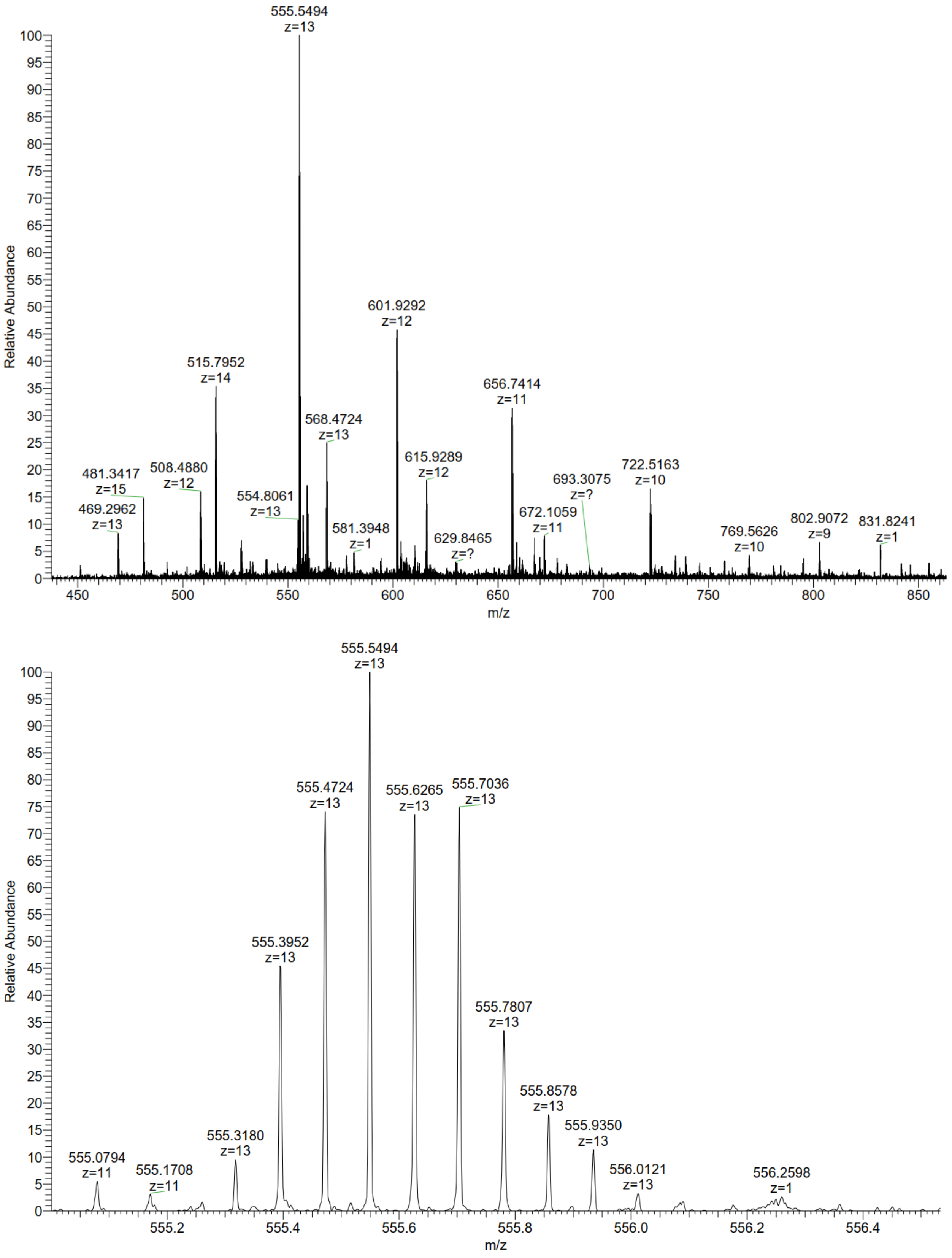

Figure S7 MS spectra of **6**.

3. Temperature-dependent UV-vis Spectra

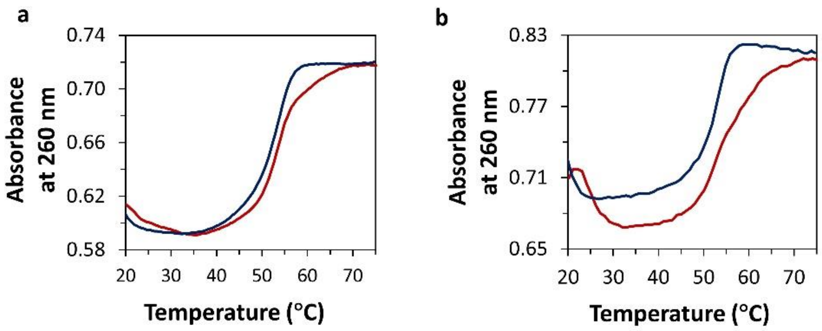

Figure S8 UV-vis absorbance at 260 nm during cooling from 75 °C to 20 °C (dark blue) and heating back to 75 °C (dark red) of (a) **3*4**, (b) **5*6** (gradient 0.5 °C·min^{-1}). Conditions: 1 µM each single strand, 10 mM sodium phosphate buffer pH 7.2, 0.03 mM spermine·4 HCl, 20 vol % ethanol.

4. Fluorescence Quantum Yield

Table S2 Mean values of four measurements of fluorescence quantum yield after self-assembly at 20°C of a solution **1*2**, **3*4**, **5*6**, and **5*6** + 6% **7**. UV-vis absorbance at 365 nm (**1*2** and **3*4**) and 345 nm (**5*6** and **5*6** + 6% **7**). Integrated fluorescence intensity between 385–720 nm (**1*2** and **3*4**) and 365–680 nm (**5*6** and **5*6** + 6% **7**). $\lambda_{ex.}$: 365 nm (**1*2** and **3*4**) and 345 nm (**5*6** and **5*6** + 6% **7**). Refractive index of 1.344 of the medium at 20 °C. Conditions: 1 µM each single strand, 10 mM sodium phosphate buffer pH 7.2, 0.03 mM spermine·4 HCl, 20 vol% ethanol.[a] addition of 0.06 µM of **7**.

Duplex	Fluorescence Quantum Yield (ΦF) after self-assembly at 20°C [%]
**1*2**	34.0±3.1
**3*4**	31.3±0.8
**5*6**	3.1±0.1
**5*6** + 6% **7**[a]	6.8±0.3

5. Cryo-EM

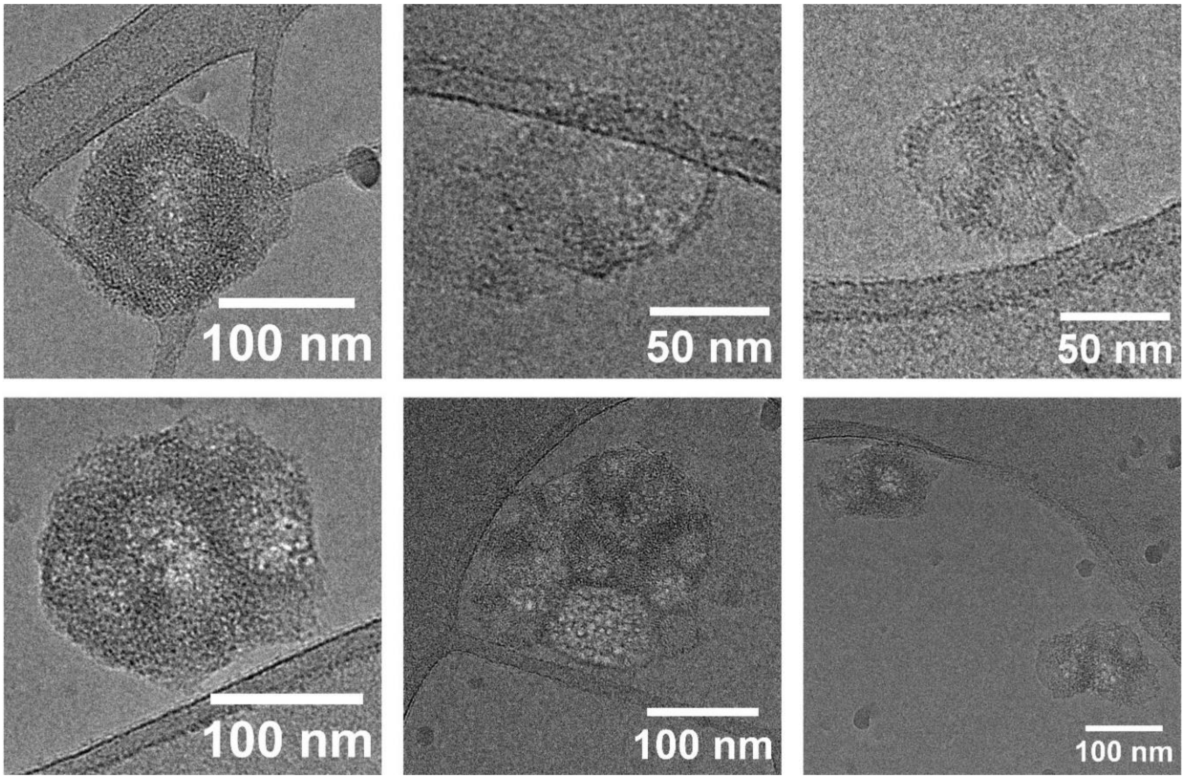

Figure S9 Cryo-EM images of **1*2**. Conditions: 1 µM each single strand, 10 mM sodium phosphate buffer pH 7.2, 0.03 mM spermine·4 HCl, 20 vol % ethanol.

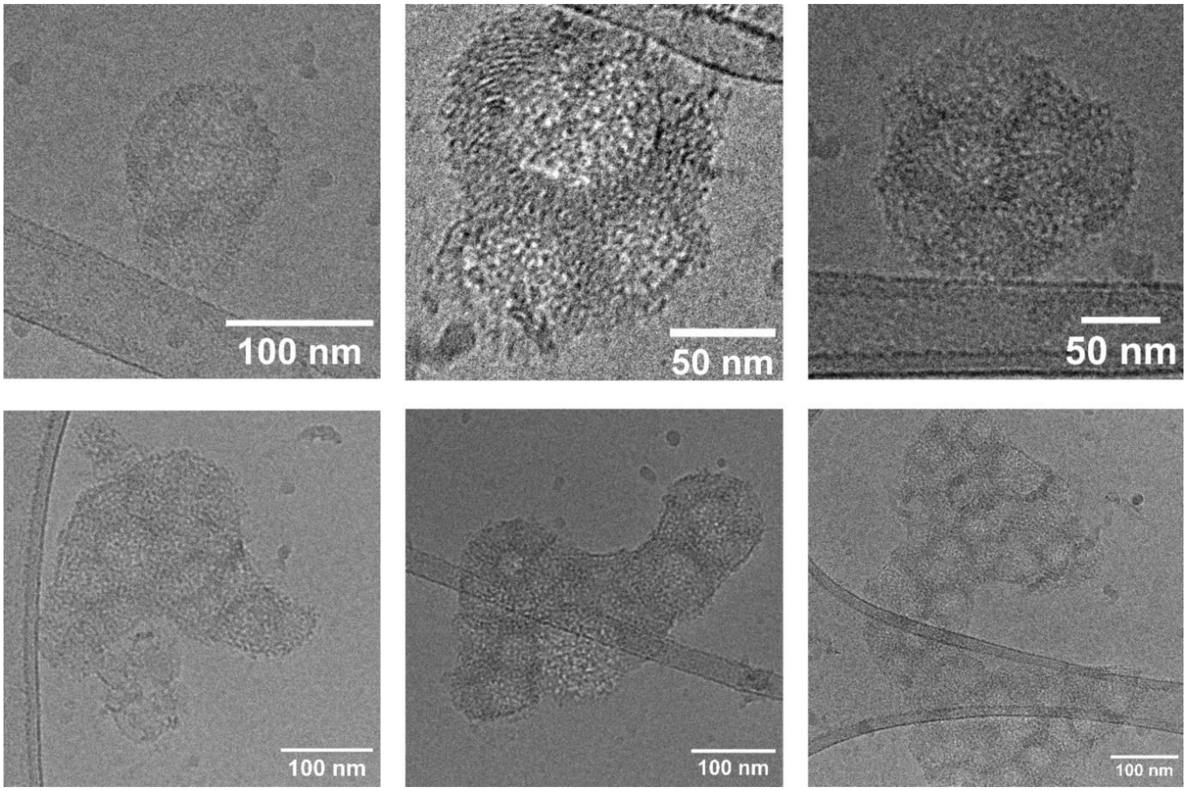

Figure S10 Cryo-EM images of **3*4**. Conditions: 1 µM each single strand, 10 mM sodium phosphate buffer pH 7.2, 0.03 mM spermine·4 HCl, 20 vol % ethanol.

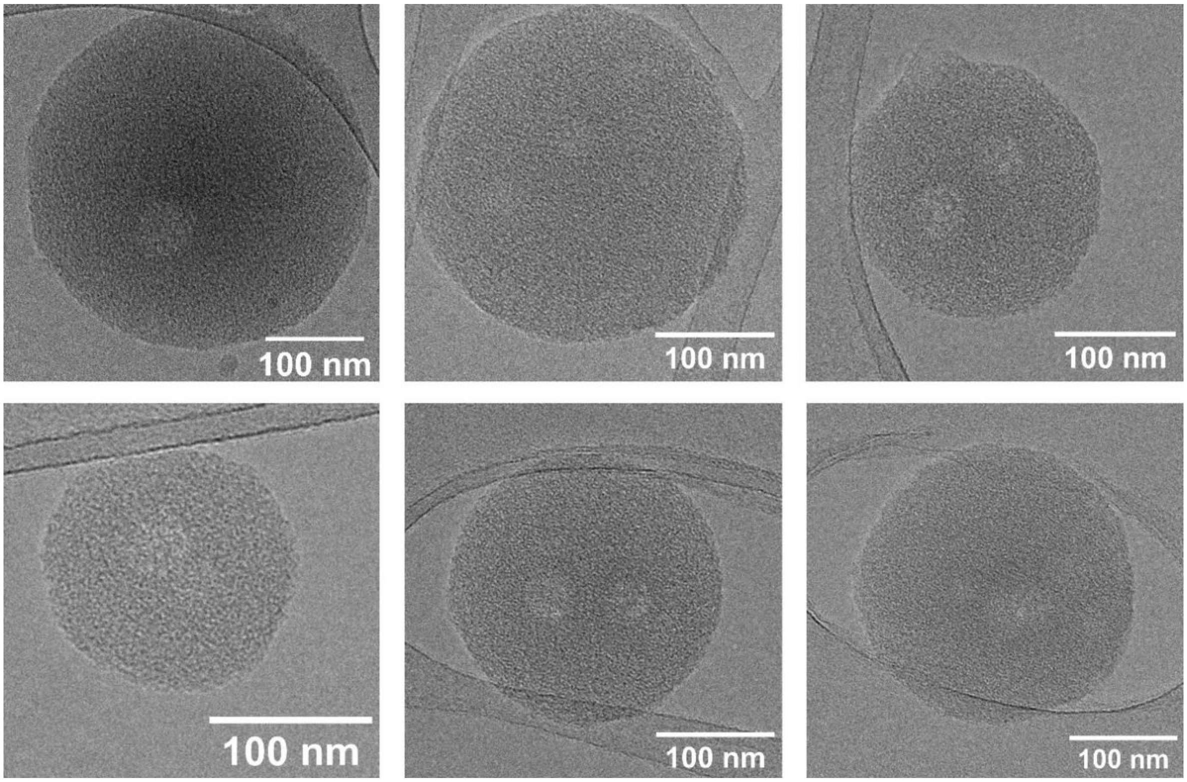

Figure S11 Cryo-EM images of **5*6**. Conditions: 1 µM each single strand, 10 mM sodium phosphate buffer pH 7.2, 0.03 mM spermine·4 HCl, 20 vol % ethanol.

6. AFM

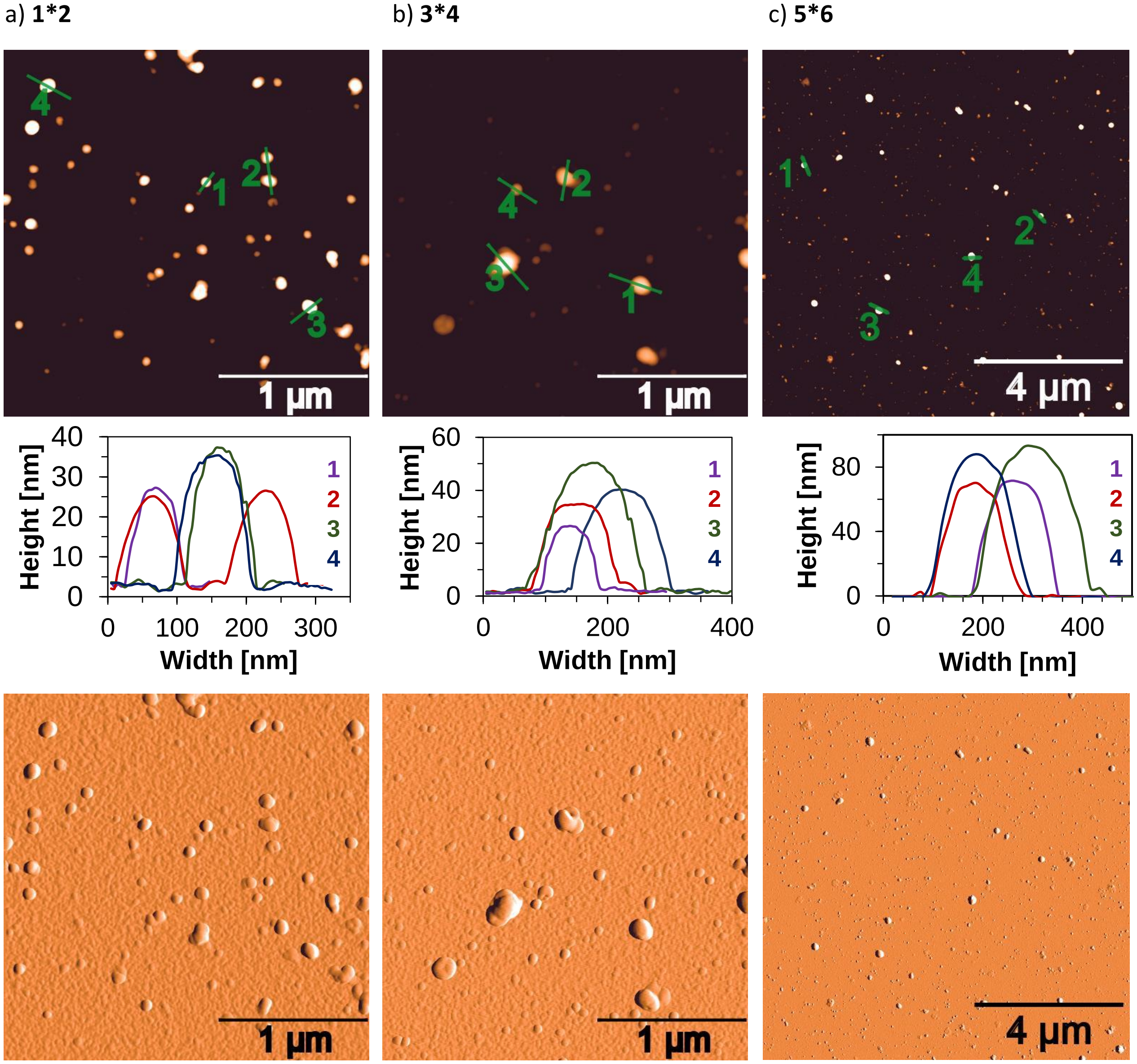

Figure S12 AFM images including cross sections (top), corresponding height profiles (middle), and deflection scan (bottom) of the self-assembled (a) **1*2**, (b) **3*4**, and (c) **5*6** on APTES-modified mica. Conditions: 1 µM each single strand, 10 mM sodium phosphate buffer pH 7.2, 0.03 mM spermine·4 HCl, 20 vol % ethanol.

7. Distance Measurements

Table S3 Summary of distances measured in cryo-EM and AFM images. The reported distances are mean values with the corresponding standard deviation and the number of measurements is indicated in the brackets.

Duplex	Cryo-EM Size Diameter (nm)	AFM Size Diameter (nm)
**1*2**	105 ± 46 (*n* = 288)	108 ± 50 (*n* = 57)
**3*4**	121 ± 29 (*n* = 68)	134 ± 47 (*n* = 63)
**5*6**	220 ± 60 (*n* = 36)	182 ± 55 (*n* = 61)

8. DLS

Table S4 Key results and measurement conditions of DLS measurement at 20°C of a solution of **1*2**, **3*4**, **5*6**, and **5*6 + 6% 7**. Conditions: 1 µM each single strand, 10 mM sodium phosphate buffer pH 7.2, 0.03 mM spermine·4 HCl, 20 vol % ethanol.

Duplex	Size Diameter (nm)	Z-Average Size Diameter (nm)	PDI	PDI width (nm)	Count Rate (kcps)	Attenuator
**1*2**	191.8 ± 59.5	172.2	0.107	56.38	11446.9	7
**3*4**	196.3 ± 66.5	174.2	0.113	58.44	16291.0	7
**5*6**	185.9 ± 62.2	166.1	0.103	53.30	21334.9	7
**5*6** + 6% **7**	188.1 ± 55.47	171.5	0.095	52.91	7779.5	8

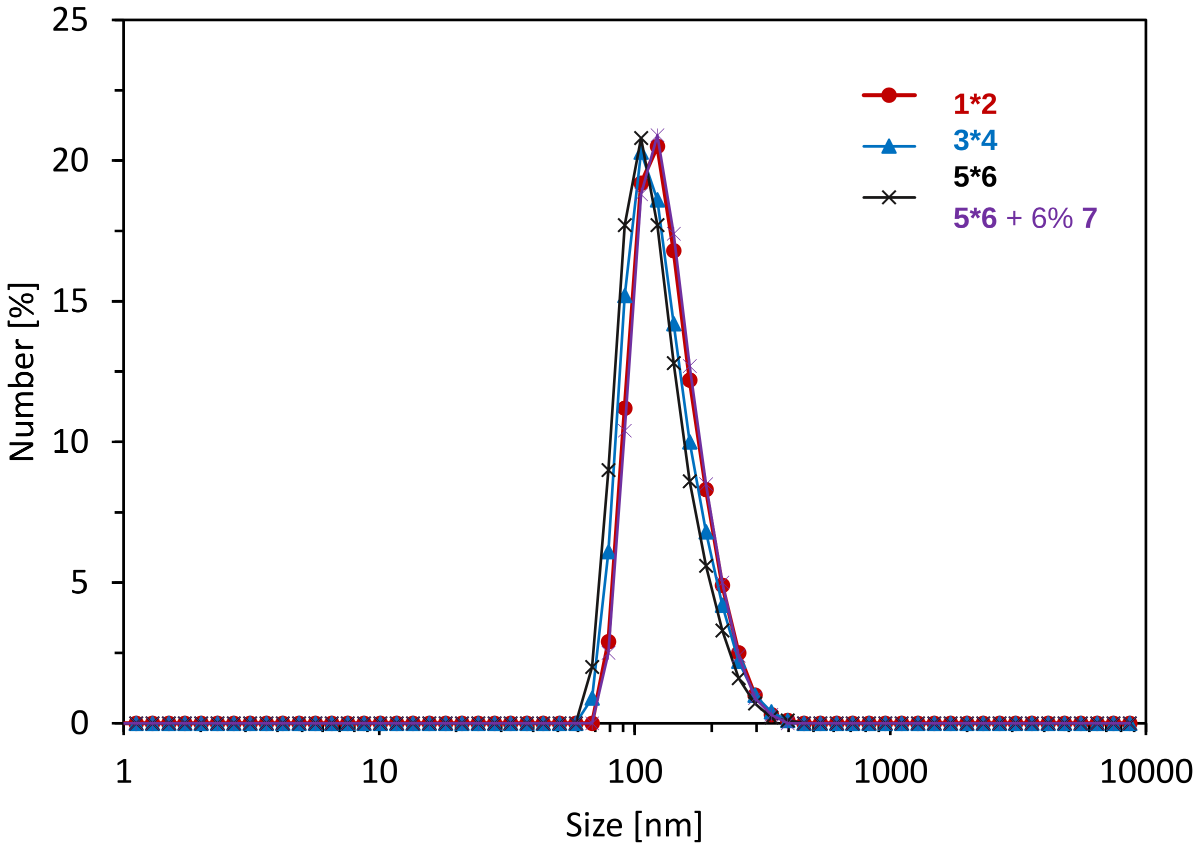

Figure S13 DLS measurement at 20°C of a solution of **1*2**, **3*4**, **5*6,** and **5*6** + 6% **7**. Conditions: 1 µM each single strand, 10 mM sodium phosphate buffer pH 7.2, 0.03 mM spermine·4 HCl, 20 vol % ethanol.

9. Fluorescence Spectroscopy

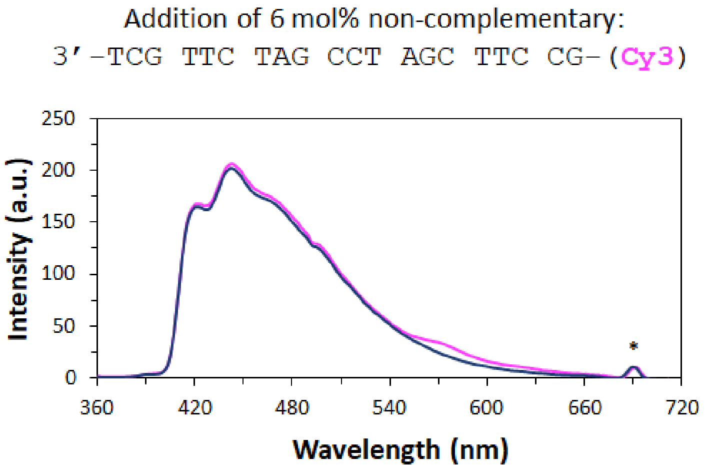

Figure S14 Fluorescence emission spectra of aggregated **5*6** at 20 °C (dark blue) and aggregated **5*6** after addition of non-complementary Cy3-modifed DNA (pink, 6 mol% per **5**) after reassembly at 20°C ($\lambda_{ex.}$ 345 nm.). Conditions: 1 µM **5** and **6** (with and without 0.06 µM 3'-TCG TTC TAG CCT AGC TTC CG-(Cy3)), 10 mM sodium phosphate buffer pH 7.2, 0.03 mM spermine·4 HCl, 20 vol % ethanol. *Second order diffraction.

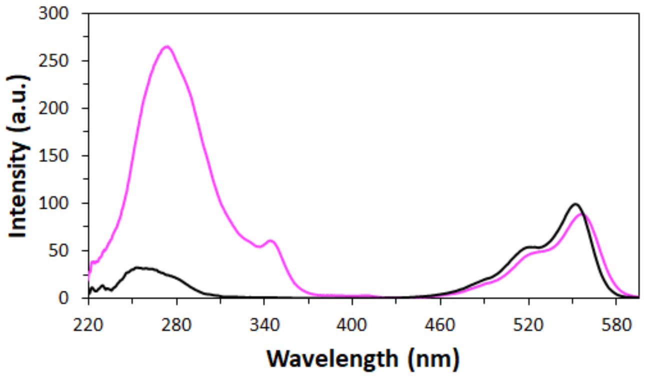

Figure S15 Fluorescence excitation spectra of 6 mol% **7** (black) and aggregated 5*6 after incorporation of Cy3-modifed **7** (pink, 6 mol% per **5**) by reassembly at 20°C (λ_{em}. 610nm). Conditions: 0.06 µM **7** (with and without 1 µM **5** and **6**), 10 mM sodium phosphate buffer pH 7.2, 0.03 mM spermine·4 HCl, 20 vol % ethanol.

10. References

1 C. D. Bösch, S. M. Langenegger and R. Häner, *Angew. Chemie - Int. Ed.*, 2016, **55**, 9961–9964.
2 H. Bittermann, D. Siegemund, V. L. Malinovskii and R. Häner, *J. Am. Chem. Soc.*, 2008, **130**, 15285–15287.
3 W. H. Melhuish, *J. Phys. Chem.*, 1961, 7.
4 S. Fery-Forgues and D. Lavabre, *J. Chem. Educ.*, 1999, **76**, 1260.
5 S. Rothenbühler, I. Iacovache, S. M. Langenegger, B. Zuber and R. Häner, *Nanoscale*, 2020, **12**, 21118–21123.
6 M. Linkert, C. T. Rueden, C. Allan, J.-M. Burel, W. Moore, A. Patterson, B. Loranger, J. Moore, C. Neves, D. MacDonald, A. Tarkowska, C. Sticco, E. Hill, M. Rossner, K. W. Eliceiri and J. R. Swedlow, *Journal of Cell Biology*, 2010, **189**, 777–782.
7 J. Schindelin, I. Arganda-Carreras, E. Frise, V. Kaynig, M. Longair, T. Pietzsch, S. Preibisch, C. Rueden, S. Saalfeld, B. Schmid, J.-Y. Tinevez, D. J. White, V. Hartenstein, K. Eliceiri, P. Tomancak and A. Cardona, *Nat Methods*, 2012, **9**, 676–682.
